# Optical characterization and through-focus performance of two advanced monofocal intraocular lenses

**DOI:** 10.1007/s00417-023-06322-8

**Published:** 2023-12-01

**Authors:** José Salgado-Borges, Anabela Borges, Isabel Ferreira, José Manuel González-Méijome, Miguel Faria-Ribeiro

**Affiliations:** 1 Clínica Oftalmológica Salgado Borges, Porto, Portugal; 2 Physics Center of Minho and Porto Universities, University of Minho, Campus de Gualtar, Ed. 6, Braga, Portugal

**Keywords:** Enhanced monofocal intraocular lens, Cataract, Power profile, Defocus curve

## Abstract

**Purpose:**

To compare the refractive power profile, subjective depth-of-field and objective optical quality of two advanced monofocal intraocular lenses (IOLs) designed to improve intermediate vision.

**Methods:**

This prospective study evaluated forty-six eyes of twenty-three patients, aged 54–68 years, binocularly implanted with two monofocal enhanced intraocular lenses (IOLs), the Tecnis Eyhance and the Physiol Isopure. Subjective through-focus visual acuity curves were obtained by placing trial lenses in front of the eye while wearing its best spherical-cylindrical correction for distance. Objective optical quality was defined as the area under the modulation transfer function, calculated from the wavefront maps measured with a high-resolution aberrometer. The optical design of both lenses was compared based on their refractive power profiles measured with the lenses immersed in saline solution.

**Results:**

Both lenses have progressive aspherical geometries, in which the sagittal power decreases rapidly from the center to the edge of the optical zone. Mean monocular through-focus curves show a best corrected distance visual acuity of − 0.02 logMAR with both lenses. Through-focus visual acuity was marginally higher for the Eyhance, with a difference of 1 letter at the defocus position of − 0.5D and 3 letters between − 1.0D and − 2.0D. Objective assessment of optical quality revealed only a difference of about 2 points in MTF area at distance.

**Conclusion:**

Both IOLs use a similar approach to improve intermediate vision. The Eyhance showed marginally better subjective performance than the Isopure at the target vergences between − 1.00D and − 2.00D, although these results did not reach statistical significance and were not replicated by the objective findings.

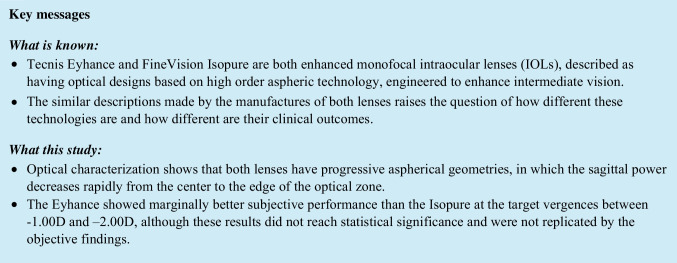

## Introduction

Monofocal intraocular lenses (IOLs) have been the standard of cataract patient care in the last decades. The first monofocal IOL to be implanted had spherical surfaces that added positive spherical aberration to the already average positive spherical aberration of the cornea [1]. The use of aspherical surfaces allowed manufacturers to create IOL designs that can balance corneal spherical aberration, from null or partial to a full compensation for improved contrast sensitivity [2].

Monofocal enhanced are a new category of IOLs created to improve the standard of cataract patient care. These IOLs are designed to extend the depth-of-field (DoFi) by a small amount, enough to provide a functional intermediate vision to cataract patients without the typical side effects from multifocal IOLs, such as glare, halos, and starburst [3].

The first enhanced IOL to be launched in the market was the Tecnis Eyhance (Johnson & Johnson Vision, Groningen, The Netherlands). This lens is described by the manufacturer as having an anterior high-order aspheric surface that deviates from its parent model, the Tecnis ZCB00, only in a limited central zone, used to create a small extension in the DoFi while maintaining the same spherical aberration correction as the rest of the TECNIS family (− 0.27 µm for a 5.15-mm aperture “in-the-bag” IOL plane) [4]. Another enhanced monofocal that was launched posteriorly is the Isopure 123 (BVI, Liege, Belgium). It is described as a non-diffractive aspherical IOL based on an aspheric polynomial technology designed to provide a distance vision similar to a monofocal, with functional intermediate vision [5]. There is no official information available for the spherical aberration correction of the Isopure.

The similar descriptions made by both manufactures raise the question of how different these technologies used to enhance intermediate vision are and how different are their clinical outcomes. The present study aims to answers these questions by conducting a comparison between these two enhanced IOLs, based on the optical characterization of both designs, subjective defocus curves, and objective image quality metrics calculated from wavefront measurements.

## Methods

This is a prospective study that evaluated 46 eyes of 23 patients of whom 12 were bilaterally implanted with the IsoPure and 11 with the Tecnis Eyhance, between May 2019 and August 2022 (*n* = 24 and *n* = 22 eyes, for the Isopure and Eyhance groups, respectively). Informed consent was obtained from all participants. Institutional clinical research ethical board committee approval was obtained (SECVS 090) from the Ethics Committee for Research in Life and Health Sciences of the Ethics Council of the University of Minho. Patients had ages between 54 and 68 years old and natural entrance pupil sizes between 3.0 and 5.0 mm, with an average value of 3.6 mm, measured under normal office photopic lighting conditions. Patient selection was carried out based on postoperative corrected distance visual acuity (CDVA) of 0.1 LogMAR or better and posterior capsule and remaining ocular medium transparency. After an initial evaluation of the anterior pole and fundus, the patient’s visual acuity was determined for different vergences (defocus curves) by adding trial lenses to the best sphero-cylindrical distance prescription, starting from + 0.50 Diopters (D) to − 3.00D, in − 0.50D steps, one eye at the time. During this process, the optotypes were randomized to prevent memorization. Finally, the last part of the evaluation consisted in the acquisition of wavefront aberrations (× 3, per eye) measured with the pyramidal high-resolution aberrometer Osiris-T (CSO, Florence, Italy). The Osiris-T offers the highest lateral resolution used by commercially available clinical aberrometers, achieving a lateral resolution of 41 μm according to the manufacturer’s specifications [6]. The high-resolution of the Osiris allows for the use of direct numerical integration of slopes for reconstruction of the wavefront, which results in a more precise description of the original wavefront data without the typical smoothing caused by modal reconstruction methods.

Objective image quality was defined as the area under the radially averaged modulation transfer function (MTFa), integrated up to 50 cycles/mm, for the same pupil diameter measured subjectively [7]. After correcting each wavefront map with its best spherical-cylindrical correction, the through-focus MTFa was calculated by adding defocus wavefronts to the distance-focused wavefront, using common Fourier optics technics based on far-field scalar diffraction, implemented in custom Matlab (MathWorks, USA) scripts. A detailed description of the procedure can be found elsewhere [8].

Refractive designs can be described very intuitively with a two-dimension power map, also known as a vergence map. A power map is a description of the lens focal power across the optical zone. In case of rotational symmetry, the power map can be replaced by a power profile, which is a two-dimensional plot that describes the change in focal power from the center to the edge of the optical zone. The ex vivo optical characterization of both lenses was made based on their sagittal power profiles measured with the NIMO TR1504 Interferometer (Lambda-X, Nivelles, Belgium), with the lenses immersed in saline solution. Only one lens of each model was measured. The NIMO TR1504 is an instrument based on the “Phase-Shifting Schlieren” technique, measuring light beam deviations with the help of Schlieren filters to calculate the power characteristics of optical lenses [9].

A sample size calculation was performed to verify the number of eyes needed. For a 95% confidence level, a clinically significant difference of 0.1 LogMAR unit for visual acuity, and a standard deviation of 0.1 LogMAR unit, the requirement was a minimum of 17 eyes in each group, with a power of 80%. The statistical analysis of the mean differences between both groups was evaluated with a linear mixed effects model, with the patient eye as a random effect to account for the inter-eye correlation due to the inclusion of both eyes of each patient [10]. All the procedures were implemented with Matlab statistical toolbox. A *p*-value of less than 0.05 was considered statistically significant for all tests.

## Results

**Fig. 1:**
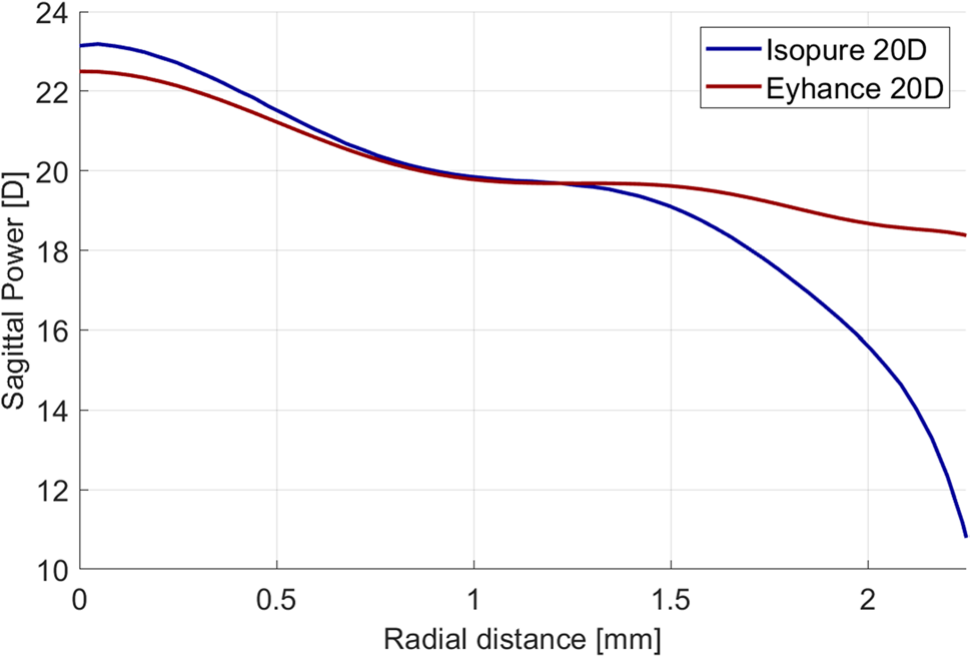
Sagittal power profiles of both monofocal enhanced IOLs immersed in saline solution, measured with the NIMO TR1504, depicting the change in sagittal power as a function of radial distance to the center of the optical zone

The sagittal power profiles of both lenses depicted in Fig. 1 show a progressive aspherical geometry, in which the sagittal power decreases rapidly away from the center of the optical zone to reach the nominal power of 20.0D at about 0.9 mm (IsoPure + 23.14D to + 20.00D; Eyhance from + 22.50D to + 20.00D, from 0 to 0.9 mm). From about 1.25 mm to the edge of the optical zone, the designs start to deviate. Compared to the Eyhance, the IsoPure shows a faster decay in power, correspondent to a higher negative spherical aberration.

**Fig. 2:**
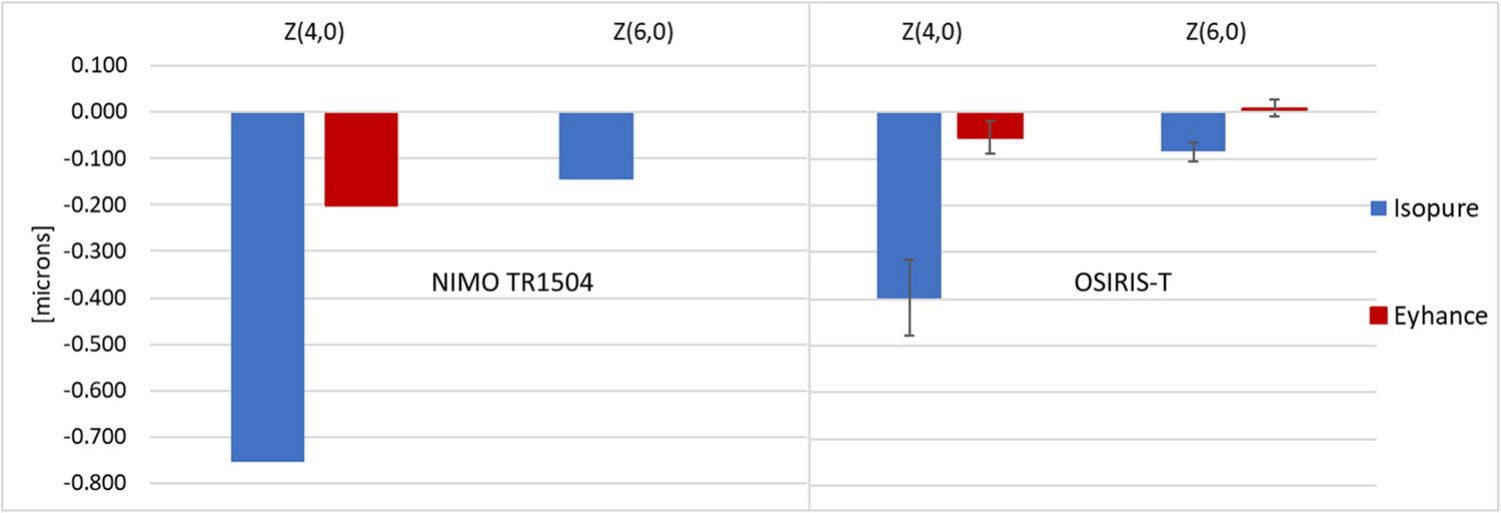
(Left) Ex vivo fourth-order (Z(4,0)) and sixth-order (Z(6,0)) Zernike spherical aberration for both IOLs, with 20.0D power, immersed in saline solution, measured with the NIMO TR1504 for a 4.50-mm optical zone. (Right) In vivo fourth- and sixth-order average Zernike spherical aberration measured with the Osiris-T, in the eyes implanted with the Isopure and the Eyhance. Coefficients were scaled from the natural pupil sizes of the patients to 5.25 mm, which corresponds to a 4.50-mm aperture in the IOL plane for an average eye. Error bars represent the 95% confidence intervals of the mean

From the power profile measured with the NIMO, it is also possible to reconstruct the wavefront error and calculate its correspondent Zernike coefficients. The left bar plots in Fig. 2 compare the ex vivo fourth- and sixth-order Zernike spherical aberration coefficients of both lenses, for a 4.5-mm optical zone, showing a more negative fourth- and sixth-order spherical aberrations for the Isopure, as expected from the power profiles in Fig. 1.

The total spherical aberration of the eyes implanted with both IOLs was calculated from the wavefront maps measured with the Osiris-T. The Zernike coefficients were scaled from their natural entrance pupil diameter to 5.25 mm, which in an average eye corresponds to an aperture of about 4.50 mm at the “in-the-bag” IOL plane. Ex vivo and in vivo spherical aberrations are depicted in Fig. 2.

**Fig. 3:**
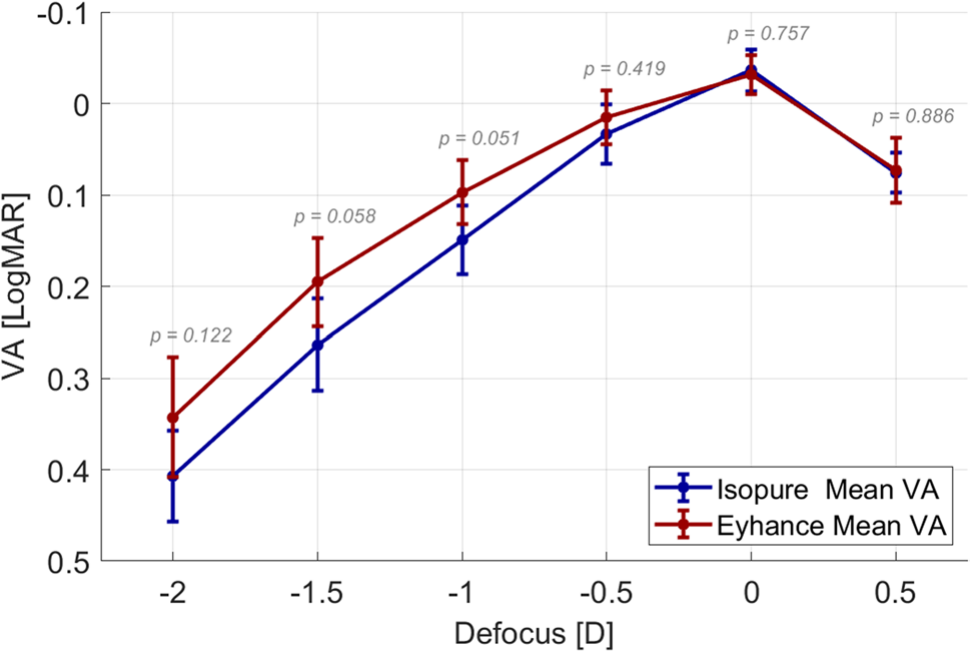
Monocular subjective visual acuity (VA) curves measured adding trial lenses to the best distance correction in steps of 0.50D. Error bars represent the 95% confidence intervals of the mean

Analysis of the mean monocular through-focus curves plotted in Fig. 3 revealed a best corrected monocular distance visual acuity of − 0.02 LogMAR with both lenses. The through-focus visual acuity was marginally higher for the Eyhance, with a difference of only 1 letter at the defocus position of − 0.50D (2 m) and of about 3 letters at the defocus positions between − 1.00D and − 2.00D (1 to 0.5 m). The difference in mean visual acuity between both lenses was not statistically significant throughout the defocus range when considering a 95% confidence level, although the differences were close to achieve statistical significance for defocus positions of − 1.00D and − 1.50D (*p*-value = 0.051 and *p*-value = 0.058, respectively).

**Fig. 4:**
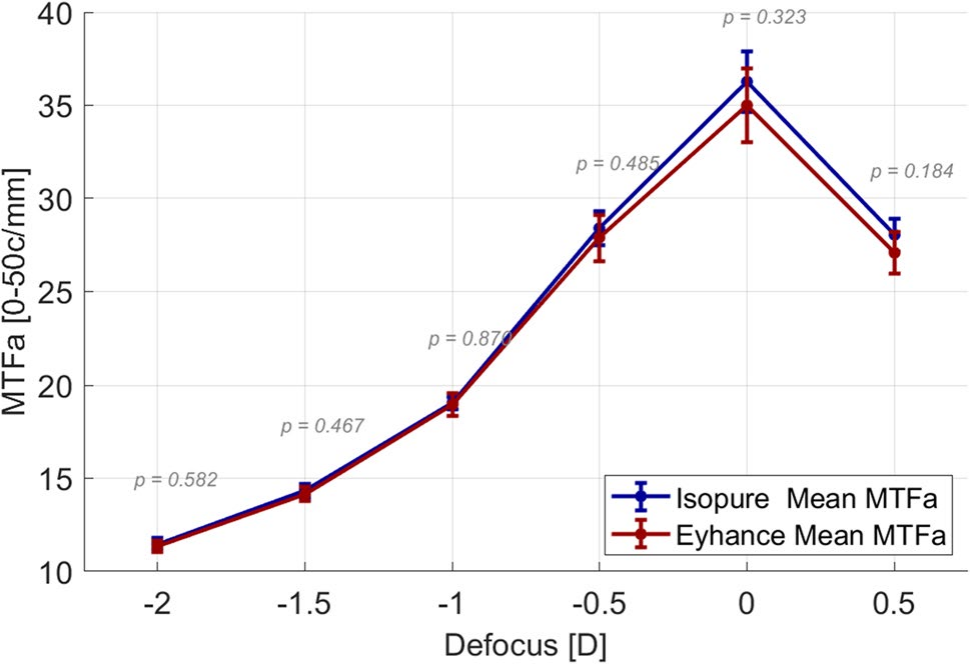
Monocular objective image quality (MTF area) defocus curves calculated from the aberrometry data measured with the Osiris-T aberrometer and Fourier optics methods. Error bars represent the 95% confidence intervals of the mean

The objective assessment of optical quality based on wavefront measurements plotted in Fig. 4 revealed only a clinically and statistically insignificant marginal difference in best corrected distance optical quality, with a higher value in MTFa obtained with the IsoPure (36.28 ± 1.62 vs. 35.01 ± 1.97, mean ± CI95%) and almost undifferentiated through-focus image quality from − 0.50D to − 2.00D defocus positions.

**Fig. 5:**
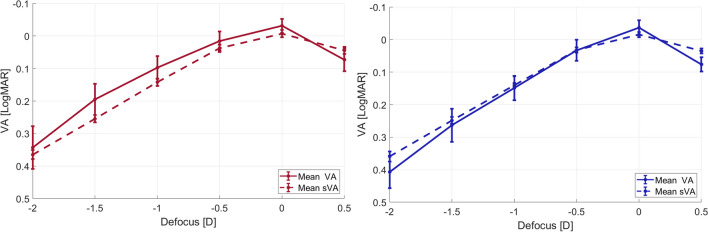
Clinical (continuous curves, VA) versus objective (dotted curves, sVA) visual acuity, for the Eyhance (left) and the Isopure (right) IOLs. Error bars represent the 95% confidence intervals of the mean

To establish a direct comparison between clinical visual acuity, the through-focus MTFa curves were converted to simulated visual acuity according to the nonlinear relation sVA = *a* × MTFa^*b*^ + *c* described by Alarcon et al. [7], with *a* = 6.27, *b* =  − 1, and *c* = -0.25, obtained from fitting clinical VA to MTF area, following the method described in Fernández et al. [11]. Results plotted in Fig. 5 compare the visual acuity measured with trial lenses with the simulated visual acuity calculated from the measured wavefronts, showing a good agreement between both subjective and objective defocus curves.

## Discussion

To achieve the clinical outcomes claimed for these monofocal enhanced IOLs, manufacturers need to deviate from the perfect monofocal optics to obtain a design that can generate a point spread function slightly more resilient do defocus than a typical monofocal design would. This is the case of both IOLs under analysis. It seems that both IOLs employ similar approaches to obtain functional intermediate vision that consists of aspheric surfaces with paraxial focal power above the nominal power that decreases very rapidly away from the center of the optical zone up to about 0.9 mm. After this point, the designs start to diverge from each other, with the Isopure showing a larger negative gradient in power that will overcompensate the average corneal spherical aberration [1]. This feature of the Isopure design is probably intended to further increase the DoFi for eyes with larger pupils. The increased negative spherical aberration in the Isopure design can be partially compensated by targeting a small myopic residual error, leading to a proportional increase of the lens central power, which will be equivalent to shift the power profile plotted in Fig. 1 up in the *y*-axis. On the other hand, if emmetropia is achieved for photopic pupil diameters, a likely consequence of this increased negative spherical aberration is that as the pupil gets larger, under low illumination levels, the Isopure design might induce a small hyperopic residual error. This effect was also measured by Azor et al. in an ISO2 optical bench setup, with an artificial cornea with a spherical aberration of + 0.27 µm for a 6-mm diameter [12]. These authors reported a hyperopic shift of + 0.20D and 0D for the Isopure and Eyhance, respectively, for an aperture diameter of 4.5 mm in the IOL plane, which corresponds to the diameter of the optical zone plotted in Fig. 1. The opposite effect, i.e., small myopic shifts, might also be expected with both lenses when the diameter of the optical zone being illuminated is around 2 mm, as reported by the same authors [12], which would be the case in an eye with an entrance pupil of about 2.4 mm. The agreement between the results reported by Azor et al. and the inferences made from the power profiles can be justified from the fact that the balance of energy at the image plane will be related to the ratio between the different refractive powers inside the area of the optical zone being illuminated [13]. For both IOL models under analysis, when the diameter of the aperture at the IOL plane is smaller than 2 mm (radius < 1 mm), light will be refracted only by the central part of the optical zone that contributes very little to distance; thus, a myopic shift occurs. This effect can either be positive or negative, depending at which distance the patient is looking at. In other words, one can infer the pupil dependence of these designs from looking at the power profile. This pupil-dependent behavior might impact the general satisfaction of patients, as their natural pupils change size under different illumination conditions. These aspects should be evaluated in future studies, through clinical quality of vision questionnaires and/or psychophysic testing.

The ex vivo fourth-order spherical aberration found agrees with the values reported by Alarcon et al. [4] for the Eyhance and the Tecnis standard monofocal (approximately − 0.22 and − 0.21 microns for a 4.5-mm optical zone, respectively), measured with the Hartman-Shack aberrometer Crystalwave (Lumetrics, Rochester, USA). The ex vivo fourth-order spherical aberration found for the Isopure is in close agreement with the value reported in Fig. 9C of its patent application [14], measured in an optical bench with a neutral spherical aberration cornea. The in vivo spherical aberration values also agree with previous results [15] that reported a more negative spherical aberration with the Isopure than with the Tecnis standard monofocal.

To our knowledge, this is the first study to conduct an objective evaluation and comparison of both the Eyhance and the Isopure IOLs. The optical characterization of both lenses in terms of its power profile can be found in the correspondent patent and patent application. The results obtained with the NIMO TR1504 seem to agree with the manufacturer’s description for the Eyhance, plotted in the patent’s Fig. 4 [16]. In the case of the Isopure, and although the patent application does not provide numerical data in the power profile of its Fig. 10A [14], the resemblance between its shape and the power profile measured with the NIMO is obvious.

The similarity obtained in the subjective results are also consistent with the similar refractive profiles measured with the NIMO TR1504, considering the average natural entrance pupil diameter of the patients (3.6 mm). Although the number of patients in the present analysis is low, the subjective through-focus performance of both the Eyhance and the Isopure is consistent with previous reports for monocular visual acuity defocus curves. For instance, Bova and Vita [15] reported mean monocular visual acuities in 42 eyes implanted with the Isopure of 0.18 and 0.28 LogMAR, at defocus positions − 1.00D and − 1.50D, respectively, which are in close agreement with the present findings (0.15 and 0.26 LogMAR, respectively). Stodulka and Slovak [17], who evaluated 36 eyes implanted with the Isopure, also reported similar mean monocular values of 0.18 and 0.30 LogMAR for the same defocus positions of − 1.00D and − 1.50D, respectively. For the Eyhance, the present results also agree with previous findings. Yangezes et al. [18] evaluated 71 eyes implanted with the Tecnis Eyhance. These authors reported mean monocular visual acuities of 0.10 and 0.20 LogMAR at − 1.00D and − 1.50D, respectively, which agree with the present findings.

In conclusion, both IOL designs use a similar approach to enhance the depth-of-field and provide functional intermediate vision. In the present findings, the Eyhance showed marginally better subjective performance than the Isopure at the target vergences between − 1.00D and − 2.00D. Although a small difference of 3 letters might approach clinical relevance, these results did not reach statistical significance and were not replicated by the objective findings.
